# Junction flow inside and around three-row cylindrical group on rigid flat surface

**DOI:** 10.1016/j.heliyon.2022.e12595

**Published:** 2022-12-24

**Authors:** Volodymyr Voskoboinick, Arthur Onyshchenko, Oleksandr Voskoboinyk, Anastasiia Makarenkova, Andrij Voskobiinyk

**Affiliations:** a Institute of Hydromechanics of the National Academy of Sciences of Ukraine, Kyiv, 03057, Ukraine; b National Transport University, Kyiv, 01010, Ukraine

**Keywords:** Junction flow, Cylindrical group, Velocity fluctuations, Integral, spectral and correlation characteristics

## Abstract

Groups of bluff bodies are widespread in nature and technology. These are the supports of bridge crossings, high-rise buildings in cities, offshore drilling and wind platforms, algae and vegetation in the seas and rivers, forests and other objects. The flow of air or water around such structures has a complex vortex and jet character and requires significant efforts in the process of scientific research to improve the environmental situation and reduce material and technical costs in the process of operating such structures. The purpose of the research is study the features of the generation and evolution of vortex and jet flows near and inside the three-row group of cylinders, which are installed on the rigid flat surface. The results of experimental studies showed that the flow around the group of cylinders had a complex unsteady nature, which is due to the interaction of vortex and jet flows typical flow elements with the three-row cylindrical group, which was located installed on the rigid flat surface. The three-row cylindrical group (31 piles with a diameter of 0.027 m) is a model of a bridge support, which was streamlined at a velocity of 0.06 m/s to 0.5 m/s (Reynolds number Re_d_=(1600–6700) and Froude number Fr=(0.04–0.18)). Visual investigations and measurements of the velocity field were carried out inside and around the three-row structure. The features of the formation and evolution of vortex and jet flows inside and near the cylindrical group were established. Integral, spectral and correlation characteristics of the velocity fluctuation field were obtained. Mean, root-mean-square values of velocity and probability density functions of velocity fluctuations integrally displayed the changes in the velocity field in the spatial and temporal domain in the junction area of grillage and plate. The power spectral densities of velocity fluctuations and mutual correlation functions made it possible to study the features of the generation of the velocity fluctuation field in the frequency domain and its interrelationships in space. It was revealed that the velocity field inside the horseshoe vortex structures was multimodal. The spectral levels of velocity fluctuations at the periphery of the quasistable horseshoe vortex structures were higher than in the cores of these structures. The highest levels of the velocity fluctuation spectra were observed in front of the second lateral cylinder where the interaction of the vortex and jet flows took place. Discrete peaks in the spectral levels of velocity fluctuations are found at the frequencies of formation of large-scale wake vortices and the frequencies of formation of small-scale vortex structures of the shear layer, which are due to the Kelvin-Helmholtz instability. It has been established that the frequency of formation of shear layer vortices is (10–40) times higher than the frequency of formation of wake vortices.

## 1 Introduction

Vortex and jet flow is a classic branch of fluid mechanics that is widely used in aero hydrodynamics and thermal physics (Devenport and Simpson, 1990; Simpson, 2001; Apsilidis et al., 2015). When the stream flows around an obstacle, which is installed on the surface, an adverse pressure gradient is formed in front of the obstacle that leads to the separation of the boundary layer. A downflow is formed along the frontal surface of the obstacle, which rushes into the junction region of the obstacle with the streamlined surface. The interaction of the separation flow and the downflow generates a system of horseshoe vortices that envelope the base of the obstacle. Horseshoe vortex structures are unstable, change their shape, number, circulation and location, interact with each other and the streamlined surface, pair and break under the action of Reynolds and viscous stresses (Rona et al., 2020; Robison et al., 2021; Qi et al., 2022; Lin and Wu, 2022). On the lateral sides of the bluff body, separated shear layers are formed. Behind the obstacle these layers roll into oppositely rotating large-scale wake vortices or vortex tubes. At the corresponding Reynolds numbers, wake vortices form a Karman vortex street. The interaction of vortex structures, jet streams and shear layers with each other and with streamlined surfaces leads to significant nonlinearity and unsteadiness of the vortex flow, intense turbulence, increased shear stresses and scour near the obstacle, which is installed on the eroded surface (Voskoboinick et al., 2016; Elahi et al., 2017). If a group of obstacles is located on the streamlined surface, then the structure of the junction flow is greatly complicated and requires significant resources for its study.

Large groups of bluff bodies are widespread in nature - these are forests, algae and vegetation in water areas, coral reefs in the seas and oceans, and much more. In such objects, a complex movement of air and water masses occurs. It is formed vortex and jet flows, which affect the microclimate and functions of plants, as well as the vital activity of living organisms that live in such arrays. Understanding the nature of turbulent flow in vegetation is important in forestry, hydrology and agriculture. The relevance of scientific research to study the features of the formation and development of flow inside and near group structures of streamlined bodies is due to the search for more sustainable methods of environmental management, improvement of ecology and the use of natural resources, the development of weather and climate forecasting systems, as well as the need to study global biogeochemical cycles (Lopes et al., 2015; Brunet, 2020; Koken and Constantinescu, 2021). For example, in channel streams, areas of vegetation improve water quality and habitat for fish. Heavy elements, suspended particles and some of the impurities and nutrients that enter the rivers are retained inside the vegetation. Fish fry hide from predators between plant stems. Therefore, landscaping and the use of aquatic vegetation are now widely used in river restoration and coastal protection projects (Chen et al., 2012; Neary et al., 2012; Nepf, 2012; Yang et al., 2016; Chang et al., 2018, 2020). Vegetated sections of the river increase flow resistance and create areas of high turbulence. In most studies, areas of aquatic vegetation are modeled as a group of cylinders (Chang and Constantinescu, 2015; Liu et al., 2017). The use of rigid vertical circular cylinders for modeling plant stems is widely used for numerical and physical modeling (Nicolle and Eames, 2011; Zong and Nepf, 2012; Chang and Constantinescu, 2015; Chang et al., 2017, 2018, 2020; Brunet, 2020). The features of the flow in such arrays are identical to those observed in the porous structures of shore protection and berthing structures or group cylindrical supports of bridge crossings and urban development with high-rise buildings.

Research results (Chang et al., 2017; Monti et al., 2020) have shown that a complex system of horseshoe vortices is formed in front of a porous cylindrical structure of moderate porosity (separation between the cylinders is of the order of the cylinder diameter). Vortices are generated both in front of each cylinder that form the group and in front of the entire structure of the porous cylinder. Intense interactions of vortex structures and jet flows are observed between the cylinders, especially between those that form the front part of the porous cylinder. The separated shear layers on the lateral sides of the porous cylindrical structure are much more intense than that of an equivalent solid cylinder. The generation and evolution of wake vortices and Karman vortex streets also have significant differences. Such differences in the features of vortex and jet flows lead to significant changes in the process and mechanism of scour for porous and solid cylinders that are located on the eroded soil, as well as the loads that such structures experience (Liu et al., 2008; Nepf, 2012; Chang et al., 2017; Yang and Nepf, 2019; Maji et al., 2020).

Complex piers, groups of piles or bluff bodies are increasingly used in the construction of bridge crossings, hydraulic structures, thermal power engineering, offshore drilling platforms and wind power stations and other industries for geotechnical, environmental, economic and resource-saving reasons (Ataie-Ashtiani et al., 2010; Coleman, 2005; Singh et al., 2020). However, the application of research results to single obstacles becomes problematic, due to the mutual influence of vortex and jet flows between the elements of the group structure, as well as shear layers, wake vortices and Karman vortex streets (Ataie-Ashtiani and Aslani-Kordkandi, 2013). This requires separate scientific studies of the flow characteristics in groups of bluff bodies.

In recent years, many studies have been carried out on the research of vortex and jet flows between single and group structures of bluff bodies and junction flows, both by numerical and experimental methods. RANS, DNS, LES and DES methods with various turbulence models k-ε, k-ω, k-g, SSD and others were used for numerical simulations (Duraisamy et al., 2019; Rona et al., 2020; Chang et al., 2020; Chen et al., 2020a, 2020b; Monti et al., 2020; Robison et al., 2021; Koken and Constantinescu, 2021; Hassan and Jalal, 2021; Zhao et al., 2022). Experimental studies of the fields of velocity, pressure, temperature and other parameters were carried out using a wide variety of sensors, measuring systems and equipment for recording, processing and analyzing data (Bateni et al., 2019; Gautam et al., 2019; Galan et al., 2019; Hamed et al., 2019; Priyanka et al., 2019; Guan et al., 2019; Karimaei and Zarrati, 2019; Zuccarello et al., 2020; Yang et al., 2020, 2021).

A large number of works have been carried out for tandem cylindrical structures (Mahbub Alam and Zhou, 2008; Palau-Salvador et al., 2008; Ataie-Ashtiani and Aslani-Kordkandi, 2013; Kim et al., 2017), three-cylinder structures (Noack et al., 2016; Zheng et al., 2016; Behara et al., 2017; Chen et al., 2018, 2020a, 2020b), four cylinders (Lam et al., 2003; Lam and Zou, 2010; Tong et al., 2014; Yang et al., 2021), six or more cylinders (Takemura and Tanaka, 2007; Galan et al., 2019). A number of studies have been carried out with large groups of bluff bodies (Zong and Nepf, 2012; Chang et al., 2017, 2018, 2020; Voskoboinick et al., 2021). The cylinders in groups were located in tandem, side-by-side, staggered configurations, or randomly (Ricardo et al., 2016; Shan et al., 2020; Ahmadianfar et al., 2021). Depending on the separation between the cylinders, several flow regimes were observed (Igarashi, 1981; Kim et al., 2017), namely, the flow around a group of cylinders as a single structure, when the distance between the cylinders was not large (L/D < 2). Quasi-stationary reattachment regime, when a shear layer that has separated from the surface of the upstream cylinder attaches to the surface of the downstream cylinder (2 < L/D < 5). The co-shedding regime formed a binary vortex street, which was characterized by an intense flow in the gap between the cylinders (L/D > 5). An unstable flow regime occurred when the flow was changed between the reattachment regime and the co-shedding regime (Mahbub Alam and Zhou, 2008).

The research aim is to study the features of the generation and evolution of vortex and jet flows near and inside a three-row group of cylinders, which are installed on a rigid flat surface.

## 2 Materials and methods

**Figure 1:**
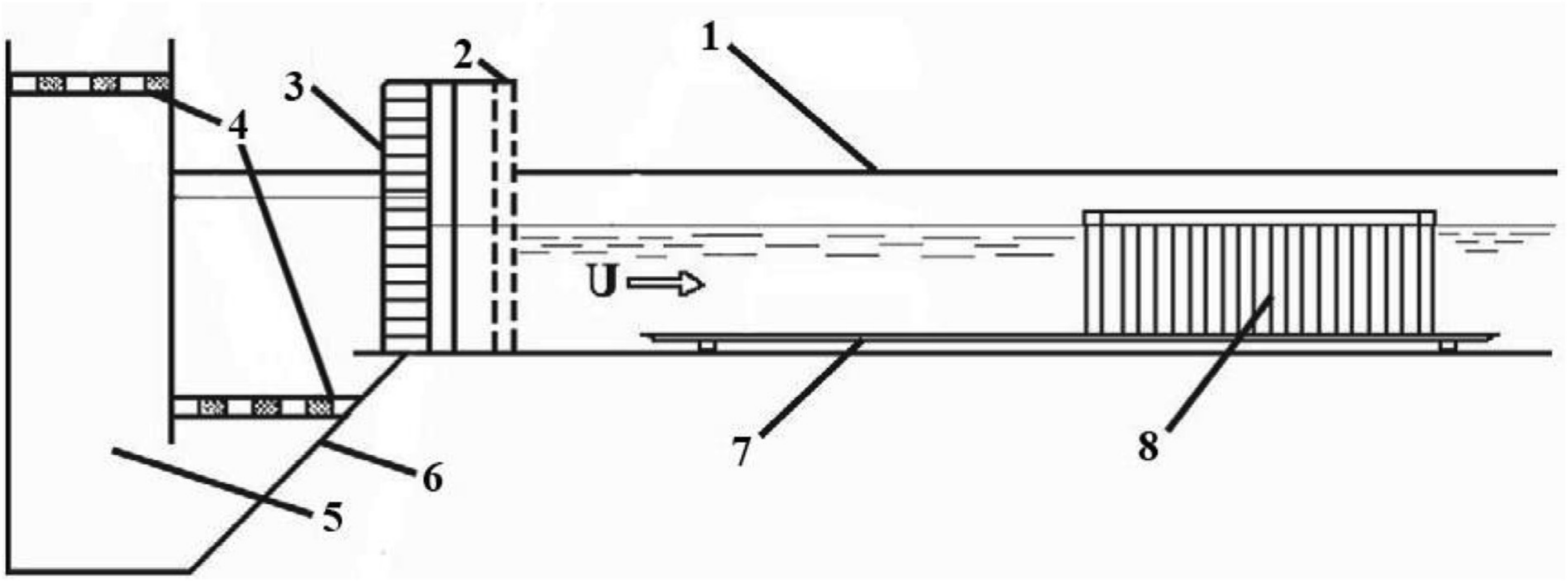
Scheme of the experimental setup. (Index 1 – the hydrodynamic flume; index 2 – the turbulizing grids; index 3 – the honeycomb; index 4 – the grate; index 5 – the settling chamber; index 6 – the confusor; index 7 – the flat plate; index 8 – the three-row cylindrical group).

Experimental studies were performed in a hydrodynamic channel in the hydraulic laboratory of the Institute of Hydromechanics of the National academy of sciences of Ukraine. The channel was approximately 14 m long, 1 m wide and 0.8 m deep. The measuring section was located at a distance of 6 m from the channel entrance. The walls of the hydrodynamic channel are made of transparent thick glass for visual research. Water was fed into the channel by means of pumps through a settling chamber, diffuser, honeycomb and grids, which directed and turbulized the flow (Figure 1). Water was discharged from the hydrodynamic channel through shields and valves that regulated the level, as well as a confusor and devices that directed the flow and absorbed its noise. Thus, the equipment of the hydrodynamic channel allowed regulating the flow velocity, its level and degree of turbulence in wide ranges. The presence of coordinate devices, streamlined holders and knives, allowed installing the experimental models, as well as measuring devices, instruments and sensors quite accurately, in accordance with the calculated values.

**Figure 2:**
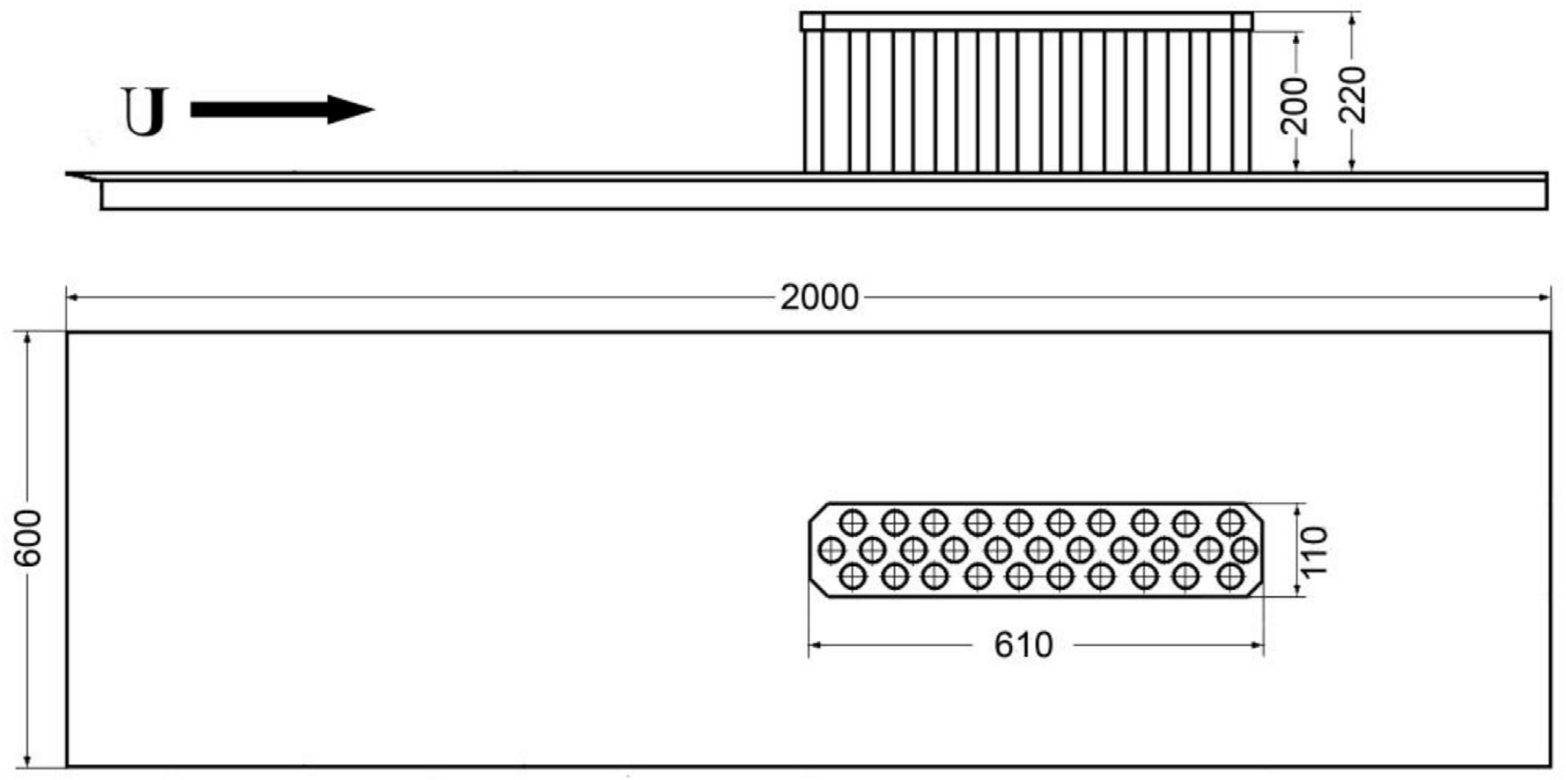
Scheme of the flat plate and the cylindrical group.

The model of a three-row cylindrical group was installed on a specially made flat plate, on which a coordinate grid was made. During visual experiments, a layer of contrast coating was applied to the plate, which was washed away by the flow. The flat plate had a width of 0.6 m and a length of 2 m (Figure 2). The three-row group model was located at a distance of 1 m from the fore part of the plate, in its middle section. The flat plate was installed along the axis and near the bottom of the hydrodynamic channel at a height of approximately 0.07 m (to reduce the influence of the boundary layer that formed above the channel bottom and the edge effects from the hydrodynamic channel walls). The water level above the plate with the group model was approximately 0.2 m and the flow velocity was regulated from 0.06 m/s to 0.25 m/s.

**Figure 3:**
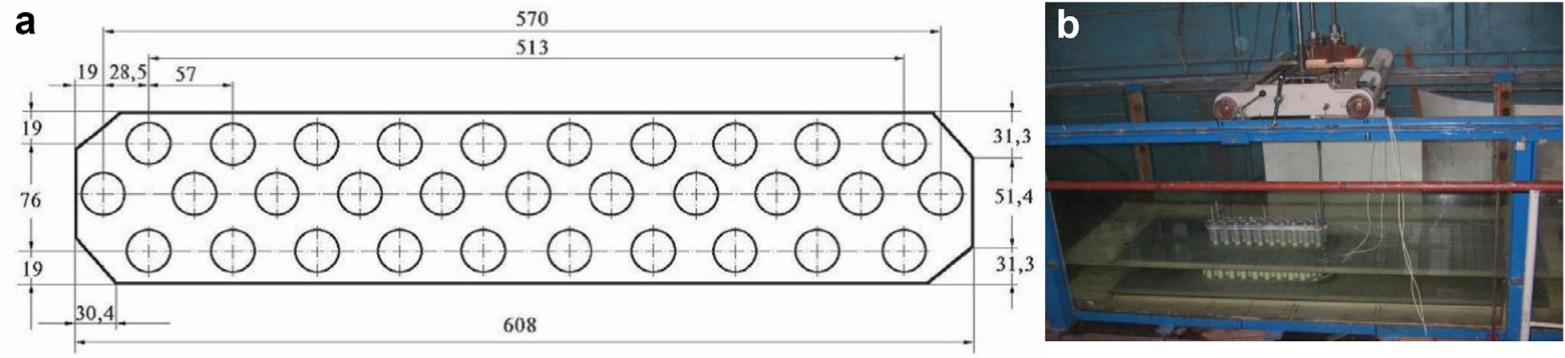
Scheme of the three-row cylindrical group (a) and photo group inside channel (b).

The model of a three-row cylindrical group consisted of 31 cylindrical piles with a diameter of d = 0.027 m (Figure 3). The piles were arranged in three rows and were arranged in a checkerboard pattern between the rows. The width of the three-row cylindrical group was 0.1 m and the length was 0.6 m. The Reynolds numbers, which were calculated from the flow velocity (U), the distance from the fore part of the plate to the first central cylinder (x), the diameter of the cylinder (d) and the kinematic viscosity coefficient (ν), were Re_x_ = Ux/ν=(4.4–18.6)·10^5^ and Re_d_ = Ud/ν=(1.6–6.7)·10^3^ For these flow velocities and flow depth (H), the Froude numbers were Fr=U/√gH=(0.04–0.18), where g is the gravitational constant. During the research, the level of flow turbulence in the hydrodynamic channel did not exceed 3%.

Estimation of spatial and temporal characteristics of vortex and jet flow near and inside the studied objects and the degree of their interaction with the streamlined surface was performed during visual studies. Visualization was performed with the help of water-soluble paints and inks, which were introduced into the study area of the flow through miniature tubes, as well as by the application of contrast coatings, which were washed away from the streamlined surface by the water flow (Voropayev et al., 2009; Voskobijnyk et al., 2016).

**Figure 4:**
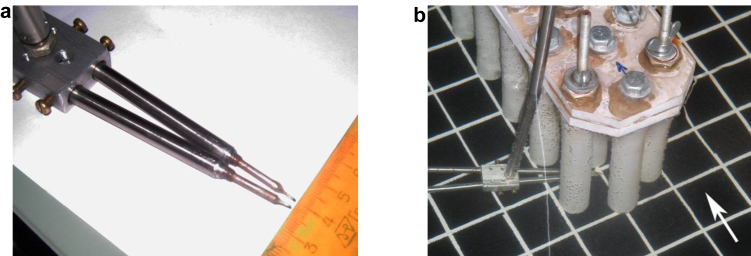
Miniature thermistor sensors of velocity (a) and location of sensors in front of the second cylinder of the three-row cylindrical group (b).

The kinematic characteristics of the junction flow were measured with the help of specially designed and manufactured miniature thermistor sensors of velocity. It is known that when a heated body or liquid is blown by a stream, convective heat exchange takes place and the heated body is cooled. The degree of cooling of a body depends both on the thermophysical properties of the interacting media and on the flow velocity. The operation of anemometers or thermistor sensors is based on the principle of heat dissipation. In our experiments, a sensitive element – thermistor used in electronics in temperature measurements was used (Voskoboinick et al., 2013, 2021). The thermistor is a semiconductor crystal with a diameter of 0.8 · 10^−3^ m with two conductive legs with a diameter of about 1 · 10^−3^ m, which were fixed in a cylindrical holder (Figure 4). When an electric current was applied, the crystal was heated, and when it was blown by flow, it was cooled. As a result, the internal resistance of the thermistor was changed and was registered. For this purpose, the thermistor was included in the bridge or half-bridge circuit, and after a change in voltage on the corresponding arms of the electric bridge, a change in temperature was recorded, which depended on the flow velocity. Then the electrical signal was amplified, filtered and fed to the control and measuring equipment and the system for collecting experimental information. The electrical signals of the thermistor velocity sensors were recorded on a four-channel precision measuring tape recorder type 7005 of the firm “Bruel & Kjaer” (Denmark).

In our experiments, we applied the technique of using two non-vector thermistor sensors in the correlation block. The sensors are installed in the holder at a fixed distance from each other (Figure 4a). The cross-correlation between the signals registered by two sensors is measured. If the signal between the sensors is not completely correlated (there is no linear connection), the cross-correlation coefficient is zero. If the signal is completely correlated (no noise), then the cross-correlation coefficient is equal to one. Intermediate values of the cross-correlation coefficient of two random signals between 0 and 1 indicate the degree of correlation of signals against the background of noise or interference. The registration of the delay time, at which the maximum of the cross-correlation coefficient is observed, allows determining the velocities of transfer of the correlation signal, since the distance between the sensors is known. The delay time sign makes it possible to find the direction of the correlation signal (Voskoboinick et al., 2013).

Visualization images were recorded by digital cameras, which were installed in different places of the measuring section of the hydrodynamic channel, as well as by digital video cameras, which had the corresponding video synchronization systems. Video and photo material was submitted to a graphics station on the basis of a personal computer, where it was processed and analyzed using special programs and algorithms (Voropayev et al., 2009; Voskobijnyk et al., 2016; Voskoboinick et al., 2013).

The electrical signals from the thermistor velocity sensors registered on the measuring tape recorder were fed to 16-channel analog-to-digital converters, and then to personal computers or to specialized two-channel spectral analyzer type 2039 of the firm “Bruel & Kjaer” (Denmark). Processing and analysis of experimental data was carried out according to standard and specially developed programs and methods with the application of probability theory, mathematical statistics and the theory of random processes (Bendat and Piersol, 2010; Blake, 1986; Vinogradnyi et al., 1989). This made it possible to keep statistics characteristics of the velocity field in the measured areas in the form of averaged components of the velocity of transfer of the correlated signal, which is due to the action of large-scale coherent vortex structures in the junction flow. The field of velocity fluctuations inside and near the three-row cylindrical group was studied using the root mean square values of velocity fluctuations and their spectral components and cross-correlations.

All measuring, monitoring, and recording equipment were tested and calibrated. The sensors were certified and checked through absolute and relative methods at specialized stands with related equipment. The measurement error of the integral and mean velocities did not exceed 4 % (with the reliability of 0.95 or 2σ), the velocity fluctuations were measured up to 6% and spectral characteristics were measured up to 2 dB in the frequency range from 0.2 Hz to 1250 Hz.

## 3 Research results

**Figure 5:**
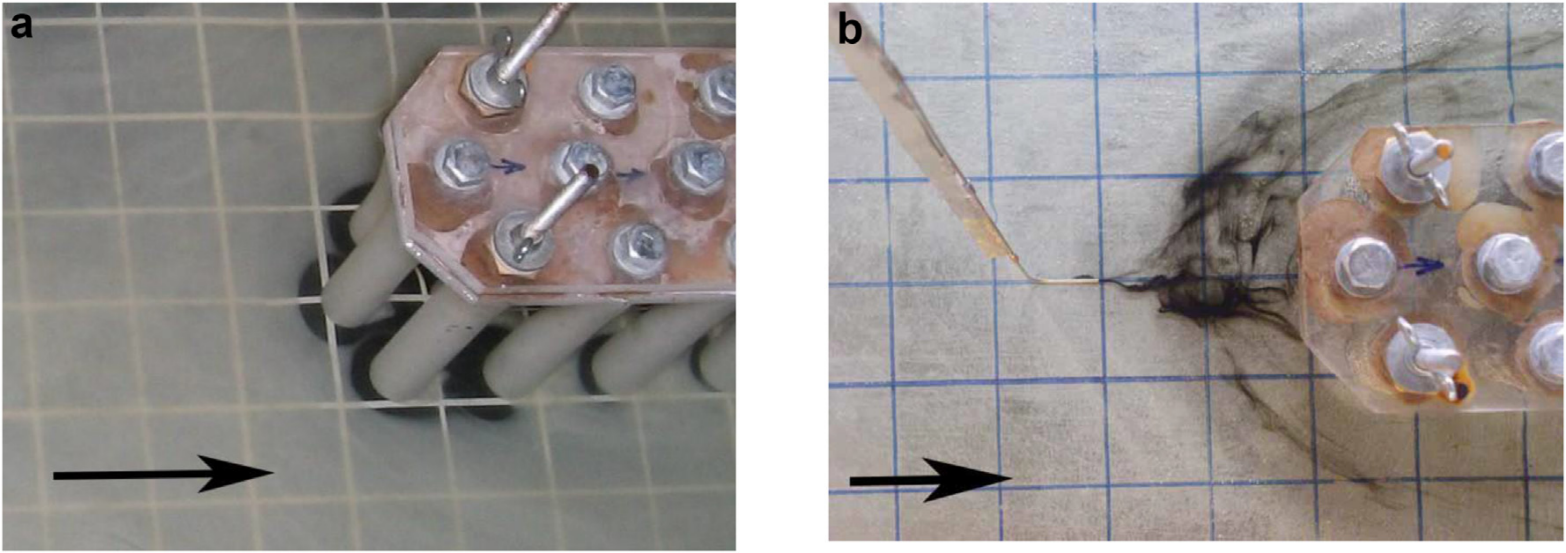
Formation of the horseshoe vortex structures in front of each cylinder (a) and formation of the large-scale system of horseshoe-shaped vortices in front of the three-row cylindrical group (b).

The results of visual experiments of a junction flow around the three-row cylindrical group showed that horseshoe vortex strictures are formed in front of the cylinders, which have some difference from the vortices that take place in the classical flow of single cylinders or small cylindrical groups. With a three-row arrangement of cylinders on a flat surface, intense horseshoe vortex structures are formed in front of each of the streamlined cylinders (Figure 5a). In addition, a large-scale system of horseshoe vortex structures, which has a low intensity, is emerging in front of the three-row cylindrical group. This vortex system covers the three-row cylindrical group as one whole structure (Figure 5b).

**Figure 6:**
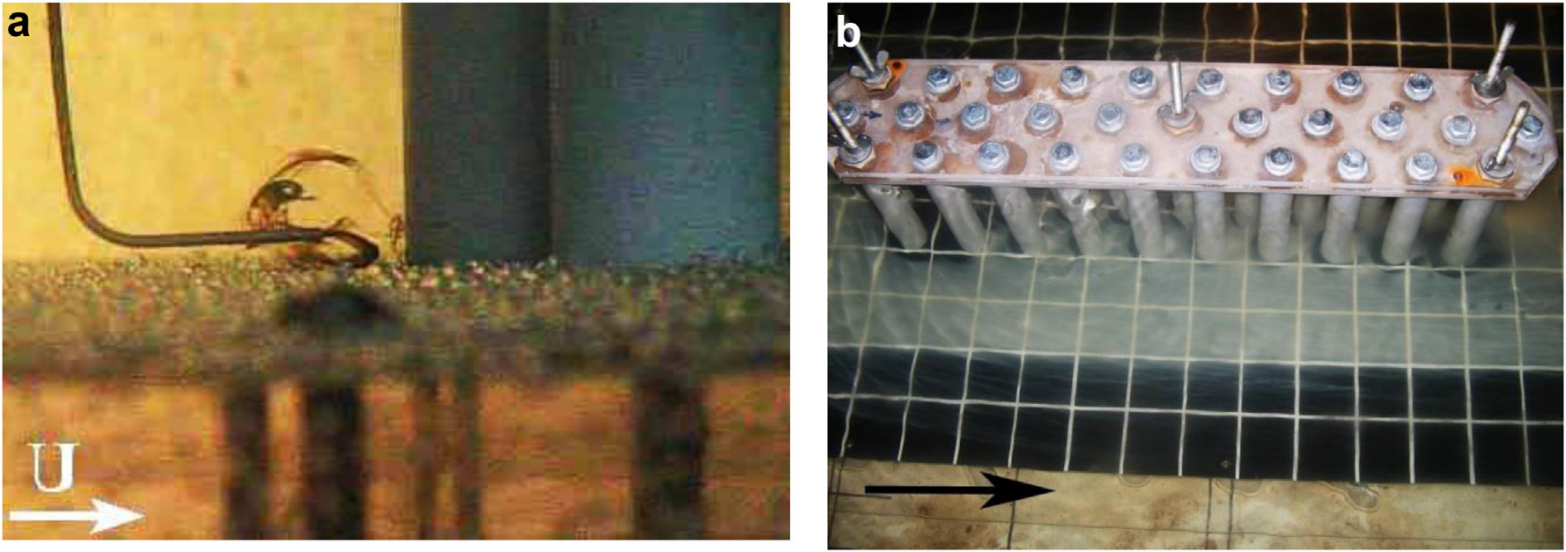
Generation of the horseshoe vortex in front of the cylinder (a) and scour of the contrast coating inside and outside the cylindrical group (b).

Scour of the contrast coating began near the frontal group of cylinders due to the downflow that took place on the frontal surface of each cylinder and the action of horseshoe vortex structures in front of each cylinder (Figures 5a and 6a). The interaction of vortex structures and a downflow led to significant wall shear stresses in the junction region of the cylinders and the rigid plate. The research results showed that the greatest erosion of the contrast coating was observed near the second lateral cylinders of the three-row design. Here, in addition to the horseshoe vortex structures and the downflow, jet flows were added, which were generated due to the separation of shear layers and the formation of wake vortices from the cylinders located upstream (Figures 5a and 6b). In the middle part of the cylindrical group, the scour of the contrast coating was minimal due to the braking effect of the group structure on the flow field. In the aft part of the three-row group, an intense erosion of the contrast coating was observed, since in this part of the structure, the flow velocity and the wall shear stresses between the cylinders were increased (Figure 6b).

**Figure 7:**
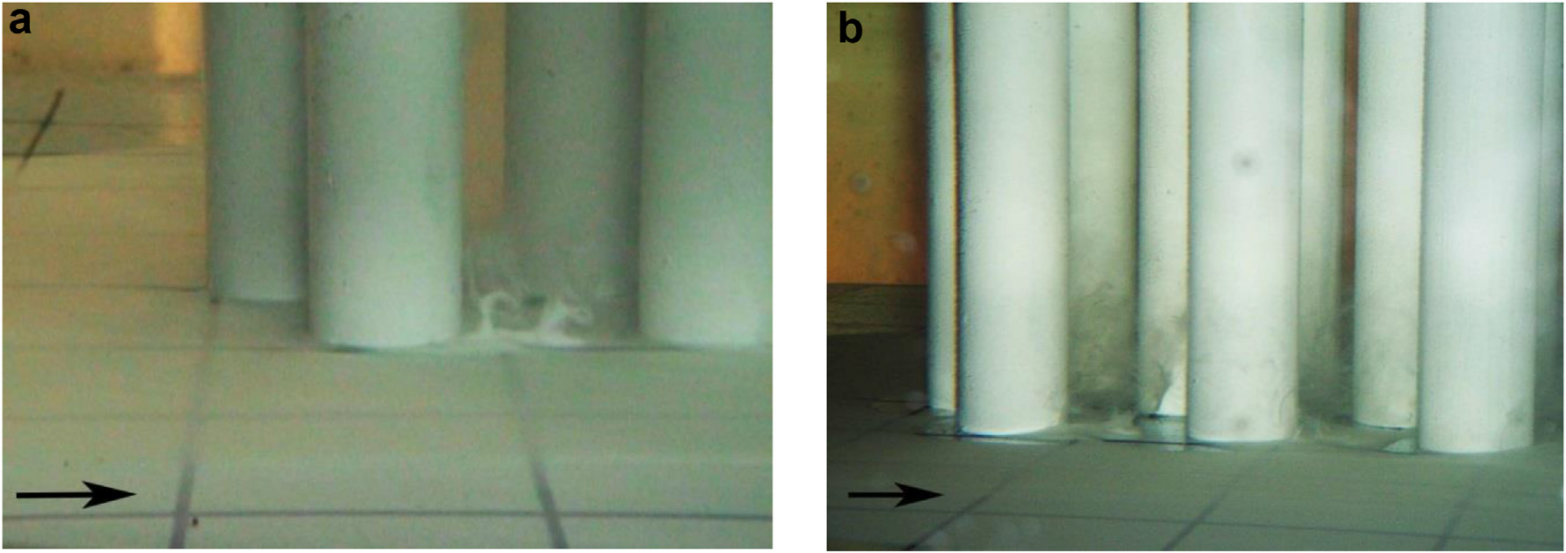
Formation of the wake vortices (a) and their destruction by jet flow (b).

Typical wake vortices are shown in Figure 7. Figure 7a shows a pair of oppositely rotating wake vortices that have separated from the first lateral cylinder. Wake vortices were formed due to the separation of shear layers from the lateral surfaces of the cylinder. Intense jet flows were generated between the cylinders, which moved outward from the cylindrical group. Wake vortices under conditions of transitional and turbulent flow behind a single transversely streamlined cylinder form a symmetric Karman vortex street (Williamson, 1996; Kirkil and Constantinescu, 2015; Garcia, 2020; Essel et al., 2021; Jiang, 2021). This vortex street is formed by separation from the sides of the cylinder of oppositely rotating large-scale wake vortices, which are carried by the flow downstream from the cylinder. In a three-row grillage, a group of cylinders is located in a checkerboard pattern (Figure 3), and intense jet flows are formed between the side cylinders of the first and second cylinders, which violate the symmetry of the movement of the Karman vortex street. The wake vortices, which are located downstream in Figure 7a were carried away by this jet stream. Figure 7b shows the moment of destruction of wake vortices by the jet flow and the rise of the contrast coating by the vertical flow behind the front lateral cylinder. Thus, a complex vortex and jet flow was formed inside the cylindrical group, which generated intense fields of velocity, shear stresses and pressure fluctuations.

**Figure 8:**
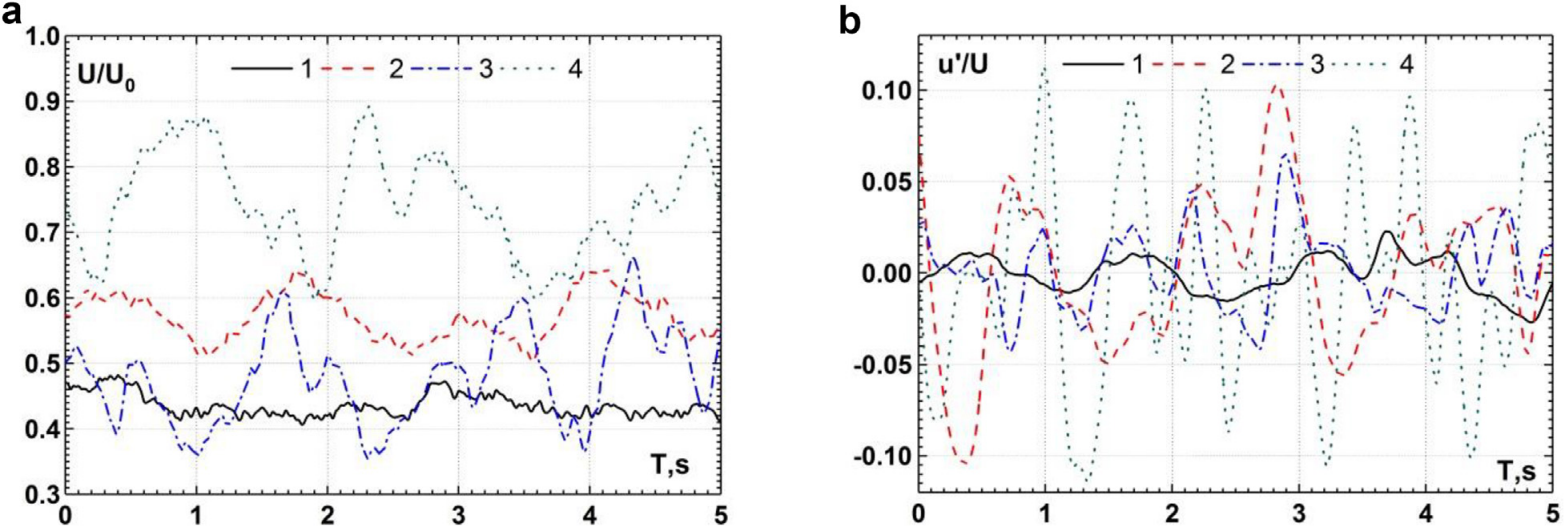
Longitudinal velocities (a) and velocity fluctuations (b) in front of the cylinders. (Curve 1 – the core and curve 2 – the periphery of the quasi-stable horseshoe vortex in front of the first central cylinder; curve 3 – the core and curve 4 – the periphery of the quasi-stable vortex in front of the second lateral cylinder).

Velocity measurements by miniature thermistor sensors made it possible to determine the integral and space-time characteristics of the velocity field inside and near the three-row cylindrical group in the region of its junction with a rigid flat surface. Figure 8 presents the results of longitudinal velocity changes and velocity fluctuations during the recording time. The study was carried out for the flow velocity U = 0.1 m/s, the Reynolds number Re_d_ = 2700 and the Froude number Fr = 0.07. Curve 1 was measured in the core of the quasi-stable horseshoe vortex in front of the first central cylinder, curve 2 was measured at the periphery of this vortex, curve 3 was measured in the core of the quasi-stable vortex in front of the second lateral cylinder, and curve 4 was measured at the periphery of this vortex. The results of the study showed that the core of the horseshoe vortex in front of the first central cylinder rotated with a linear velocity of about 0.41U, and its periphery rotated with a linear velocity of about 0.57U. The horseshoe vortex in front of the second lateral cylinder rotated at a linear velocity in the core of 0.53U and at the periphery of 0.77U. The linear velocity of the quasi-stable horseshoe vortex is higher in front of the second lateral cylinder than in front of the first central cylinder due to the influence of the jet flow between the first and second lateral cylinders. The results of the study showed that at the peripheries of the horseshoe vortex structures, the averaged and fluctuation components of the velocity are several times higher than in them cores.

**Figure 9:**
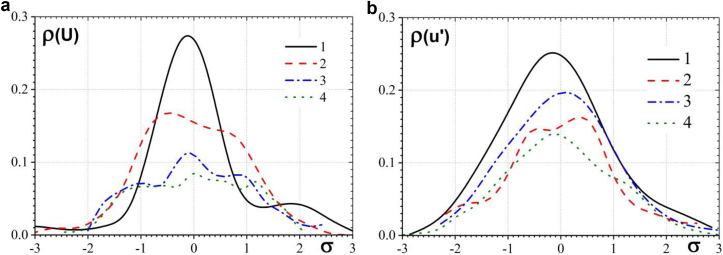
Probability density functions of the longitudinal velocities (a) and the velocity fluctuations (b) in front of the cylinders. (Curve 1 – the core and curve 2 – the periphery of the quasistable horseshoe vortex in front of the first central cylinder; curve 3 – the core and curve 4 – the periphery of the quasistable horseshoe vortex in front of the second lateral cylinder).

Figure 9 presents the results of measurements of the probability density functions of the velocity in the junction region of the cylinders with a rigid flat surface. Figure 9a shows the probability density functions of the average velocity change in front of the first central cylinder and the second lateral cylinder of a three-row structure. Figure 9b presents the probability density functions for velocity fluctuations. Curve 1 was measured in the core and curve 2 was measured at the periphery of the quasistable horseshoe vortex in front of the first central cylinder. Curve 3 was measured in the core and curve 4 was measured at the periphery of a quasistable horseshoe vortex in front of the second lateral cylinder. Changes in the velocity probability density functions are presented depending on the variances of the corresponding variables. The research results have shown that the distribution law of the probability of the values of the averaged velocity in front of the first central cylinder was close to the Gaussian or normal distribution law. However, the maximum of the probability density function was slightly shifted towards negative values of the averaged velocity. The probability of negative values of changes in the velocity of large amplitude prevailed in the field of the averaged velocity at the periphery of the horseshoe vortex. However, in the region of positive variances, an increase in the probability density function was also observed. This indicated that oscillating velocity changes were superimposed on the random velocity field. These changes were caused by oscillations of a horseshoe vortex structure in front of the central cylinder in the region of the cylinder-plate junction. A similar bimodal (double-peaks) process was observed in (Devenport and Simpson 1990; Paik et al., 2007; Kirkil and Constantinescu 2015; Jenssen and Manhart, 2020) during the interaction of vortices in a turbulent horseshoe vortex structure. As a result, two quasi-stable modes of the field of average velocities were found, namely, the backflow mode and the zero-flow mode. In the backflow mode, the flow inside the separated boundary layer rushed from the cylindrical surface towards the direction of the main flow under the horseshoe vortex structure in the form of a near-wall jet. In the zero-flow mode, the flow had a zero mean longitudinal velocity and was directed vertically or along the normal from the streamlined surface on which the bluff body was installed. Devenport and Simpson (1990) shown that bimodal coherent oscillations of a vortex flow generated intense shear stresses, which were almost an order of magnitude higher than in an approaching turbulent boundary layer. These bimodal low-frequency oscillations were due to the interaction of the downflow with large-scale vortices of the horseshoe vortex structure. The bimodal dynamics of the velocity field inside the turbulent horseshoe vortex structure was observed in front of the frontal wall in the plane of symmetry of the cylinder (for polar angles less than 70°).

The changes in the average velocity in front of the second lateral cylinder had a trimodal character (see curves 3 and 4 in Figure 9a). The appearance of the third mode was due to the influence of the jet flow, which was formed between the first and second lateral cylinders. It should be noted that three modes of transient flow, which was observed during the formation and interaction of vortices in a turbulent horseshoe vortex structure, were discovered in (Apsilidis et al., 2015; Chen et al., 2017).

The research results of the probability density functions of velocity fluctuations, which are shown in Figure 9b, showed that the field of velocity fluctuations in the region of formation and evolution of horseshoe vortex structures in front of the first central cylinder and in front of the second lateral cylinder had a more random character. Thus, the dependences of the probability density functions were unimodal (except for the field of velocity fluctuations at the periphery of the quasistable horseshoe vortex in front of the first central cylinder), which is characteristic of random processes with a Gaussian probability distribution (Bendat and Piersol 2010, Mavrakakis and Penzer, 2021).

**Figure 10:**
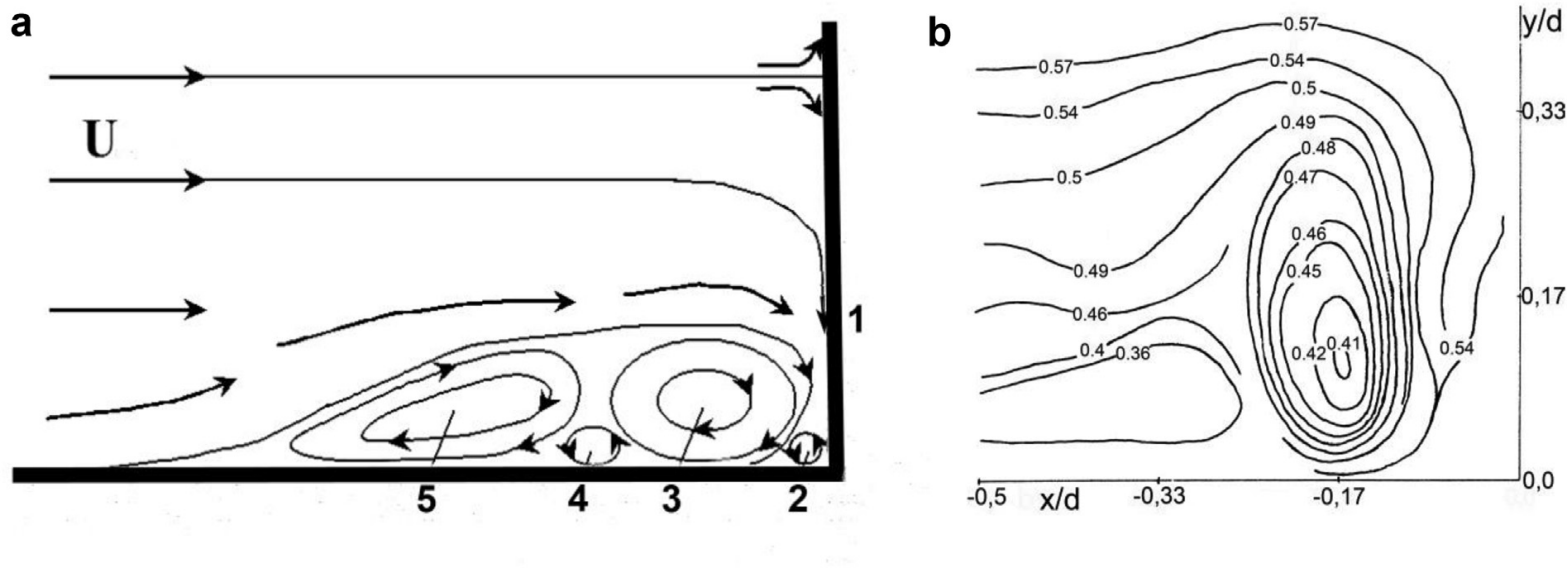
Scheme of the formation of horseshoe vortex structures (a) and the velocity field (b). (Index 1 – the downflow; index 2 – the angular horseshoe vortex; index 3 – the quasi-stable large-scale horseshoe vortex that bends around the first central cylinder; index 4 – the secondary counter-rotating vortex; index 5 – the large-scale vortex structure that surrounds the three-row design).

The results of visualization and measurements of the averaged values of the velocity made it possible to present a diagram of the formation of a horseshoe vortex structure and the field of averaged velocities in front of the first central cylinder of the group structure, which are shown in Figure 10. Figure 10a shows a diagram of the formation of the system of horseshoe vortex structures in front of the first central cylinder. Index 1 denotes the downflow, index 2 denotes the angular horseshoe vortex, index 3 denotes the quasi-stable large-scale horseshoe vortex that bends around the first central cylinder, index 4 denotes the secondary counter-rotating vortex and index 5 denotes the large-scale vortex structure that surrounds the three-row design. These vortex structures interact with each other and with the streamlined surface, oscillate in space and time, combine and collapse under the action of the junction flow(Dargahi 1989; Simpson 2001; Yang et al., 2021). As shown by the results of studies on eroded soil (Sumer et al., 2005; Yang et al., 2020; Voskoboinick et al., 2016, 2021), the large-scale vortex in front of the cylinder of the group structure (index 3 in Figure 10a) forms a local scour of the soil. Global scour of the soil around the group of cylinders is formed by the large-scale horseshoe vortex structure, which is indicated by index 5 in Figure 10a and shown in Figure 5b.

Figure 10b presents the measurement results of the average velocities in front of the first central cylinder. The results are shown as lines of equal average velocities relative to the current velocity. It was found that the core of the quasi-stable horseshoe vortex in front of the first central cylinder was at a distance of about 0.17 d from the cylinder and about 0.14 d from the rigid flat surface of the streamlined plate. A downstream flow along the frontal surface of the cylinder generated the quasi-stable horseshoe vortex and this vortex indicated by index 3 in the scheme of Figure 10a. Quasi-stable large-scale horseshoe vortex 5 in Figure 10a is formed as a result of separation of the boundary layer in front of the frontal surface of the cylinder and has core coordinates x≈−0.33d and y≈0.12d, as shown in Figure 10b.

**Figure 11:**
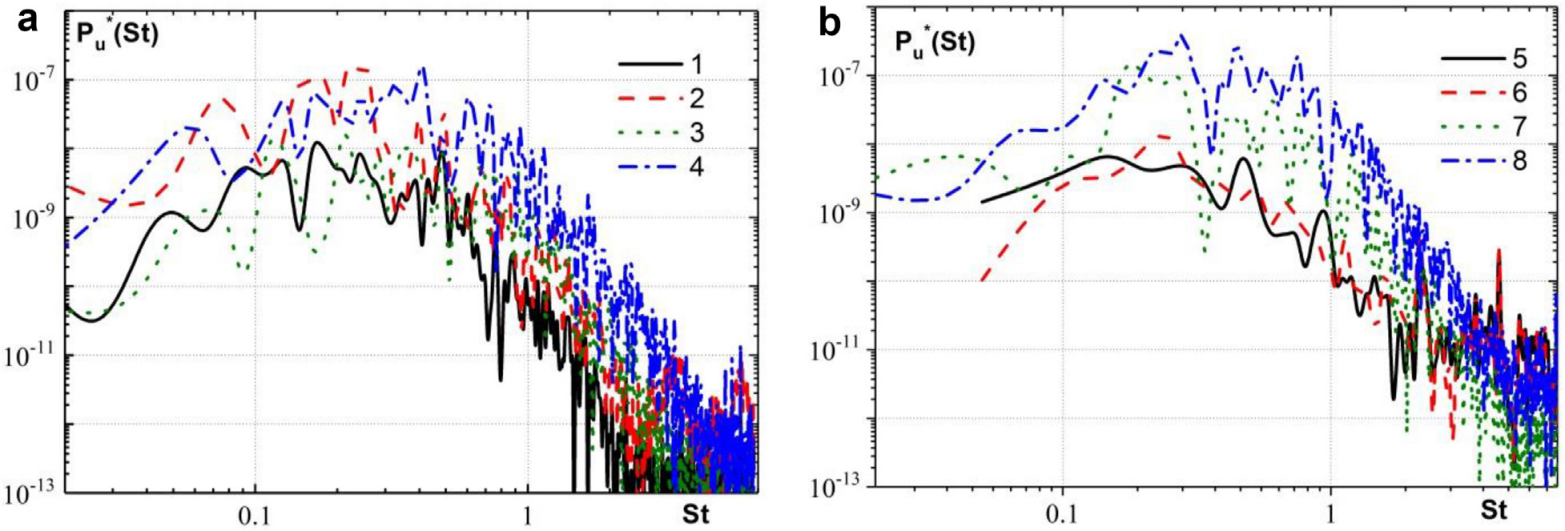
Spectral power density of the pressure fluctuations in front of the first group of cylinders (a) and in front of the second group of cylinders (b). (Curve 1 – the core and curve 2 – the periphery of the horseshoe vortex in front of the first central cylinder; curve 3 – the core and curve 4 – the periphery of the horseshoe vortex in front of the first lateral cylinder; curve 5 – the core and curve 6 – the periphery of the horseshoe vortex in front of the second central cylinder; curve 7 – the core and curve 8 – the periphery of the horseshoe vortex in front of the second lateral cylinder).

The spectral characteristics of the velocity fluctuation field in the region of the formation of quasi-stable horseshoe vortex structures in front of the cylinders of the three-row group structure are shown in Figure 11. Here the power spectral densities of the velocity fluctuations are presented depending on the Strouhal number St=fd/U. The spectra are normalized in the following form $P_{U}^{*}\left(f\right)=P^{2}\left(f\right)d/Uq^{2}$, where $q=\rho U^{2}/2$ is the dynamic pressure and ρ is the density of water. Figure 11a shows the measurement results of the power spectral densities of the velocity fluctuations in front of the first central cylinder and in front of the first lateral cylinder. Curve 1 was measured in the core and curve 2 was measured at the periphery of the horseshoe vortex in front of the first central cylinder. Curve 3 was measured in the core and curve 4 was measured at the periphery of the horseshoe vortex in front of the first lateral cylinder. The research results showed that the maximum levels of velocity fluctuations in the core of the quasistable horseshoe vortex in front of the first central cylinder were observed at a frequency of f = 0.64 Hz, which corresponded to the Strouhal number of St≈ 0.17. The maximum values of the spectral levels of velocity fluctuations at the periphery of this vortex were observed at a frequency of f = 0.93 Hz or St≈ 0.25. The maxima of the spectra in front of the first lateral cylinder were observed in the core of the horseshoe vortex structure at a frequency of f = 0.78 Hz or St≈ 0.21, and at the periphery of this vortex, the maxima of spectral levels were observed at a frequency of f = 1.52 Hz or St≈ 0.41. An increase in the frequency of the maximum spectral levels of velocity fluctuations was due to the fact that near the first lateral cylinder there was an intense interaction of the large-scale horseshoe vortex structure, which enveloped the three-row structure, with the local quasi-stable horseshoe vortex, which was formed in front of the first lateral cylinder. The large-scale horseshoe vortex went around the group of cylinders, decreased in diameter and rotated at a higher velocity, which increased the frequency of velocity fluctuations in front of the second lateral cylinder, especially at the periphery of the quasi-stable horseshoe vortex. Along with this, the intensity of velocity fluctuations in the high-frequency region of the spectrum was increased at the periphery of this vortex (see, curve 4 in Figure 11a), which were generated by small-scale sources of the fluctuations (Voskoboinick et al., 2016). The increase in the spectral components of velocity fluctuations in the frequency range f =(15–22) Hz or St =(4–6) is due to the formation and oscillations of small-scale vortices of the shear layer, which was separated from the side surfaces of the cylinder.

Figure 11b shows the results of the measurements of the power spectral densities of the velocity fluctuations in front of the second central cylinder and in front of the second lateral cylinder. Curve 5 was measured at the core and curve 6 was measured at the periphery of the horseshoe vortex in front of the second central cylinder. Curve 7 was measured at the core and curve 8 was measured at the periphery of the horseshoe vortex in front of the second lateral cylinder. The research results showed that the maximum levels of velocity fluctuations in the core of the quasistable horseshoe vortex in front of the second central cylinder were observed at a frequency of f = 0.56 Hz, which corresponded to the Strouhal number of St≈ 0.15. The maximum values of the spectral levels of velocity fluctuations at the periphery of this vortex were observed at a frequency of f = 0.89 Hz or St≈ 0.24. The maxima of the spectra in front of the second lateral cylinder were observed in the core of the horseshoe vortex structure at a frequency of f = 0.67 Hz or St≈ 0.18, and at the periphery of this vortex, the maxima of spectral levels were observed at a frequency of f = 1.04 Hz or St≈ 0.28. The Kelvin-Helmholtz instability of shear layer separation generates tonal rises in the spectra of velocity fluctuations at a frequency f =(15–19) Hz or St =(4–5), as shown in Figure 11b.

The research results showed that the spectral levels of velocity fluctuations at the periphery of the horseshoe vortex structures, which were generated in front of the streamlined surfaces of the cylinders of the group structure, were significantly higher than in the cores of these vortex structures. However, before the second central cylinder, which was located in the near wake of the first central cylinder, the spectral levels of the velocity fluctuations had comparable values. The highest values of the spectral power densities of the velocity fluctuations were observed in front of the second lateral cylinder, where, along with the horseshoe vortex structure, the jet flow took place. It was found that the maximum values of the spectral levels of velocity fluctuations were observed at higher frequencies at the periphery of the horseshoe vortex structures than in their cores.

Hydrodynamic instabilities in the form Kelvin-Helmholtz instability and Karman instability are formed in the case of a supercritical transverse flow around cylinders of circular (${Re}_{c}$ >1300) or square (${Re}_{c}$ >1000) cross section (Williamson, 1996; Prasad and Williamson, 1997; Brun et al., 2008; Franceschini et al., 2022). The Kelvin-Helmholtz instability is formed as a result of separation of the boundary layer from the streamlined surface of the cylinder and the formation of a shear layer, which is similar to the mixing layer of the jet flow (Sadr and Klewicki, 2003; Vanierschot et al., 2021). The Karman instability is formed in the wake of bluff bodies. Small-scale vortices of the shear layer interact with each other with distance from the streamlined surface of the cylinder, pair and fold into counter-rotating large-scale vortices in the near wake of the cylinder (Ahmed and Wagner, 2003; Rajagopalan and Antonia, 2005; Jiang, 2021). These large-scale vortices are periodically separated from the cylinder and, under appropriate conditions, form a Karman vortex street. In the far wake of the cylinder, the Karman vortex street is transformed into a secondary vortex street, where the secondary vortices have a larger spatial scale than the primary Karman vortices (Thompson et al., 2014; Dynnikova et al., 2016; Jiang and Cheng, 2019; Jiang, 2021). The interaction between the Kelvin-Helmholtz instability and Karman instability causes the appearance of frequency modulation of the wake turbulent flow behind bluff bodies. As the research results show, the ratio of the frequency of formation of small-scale shear-layer vortices to the frequency of generation of large-scale wake vortices ($f_{SL}/f_{V}$) is varied from 3 to >100 depending on the Reynolds number and the cross-sectional shape of the cylinder. It was established in (Bloor, 1964) that the ratio of these frequencies is proportional to $\sqrt{{Re}_{d}}$, for the flow around a cylinder in the range of Reynolds numbers $400<{Re}_{d}<250000$. In the works (Prasad and Williamson, 1997; Rajagopalan and Antonia, 2005), power-law dependences on the Reynolds number $f_{SL}/f_{V}=N{\left({Re}_{d}\right)}^{n}$ were also proposed. In this case, the exponent (n) is varied from 0.5 to 0.87, and the coefficient of proportionality (N) is varied from 0.02 to 0.1. In research (Rajagopalan and Antonia, 2005) a dependence $f_{SL}/f_{V}=0.029{Re}_{d}^{0.65}$ was obtained for the transverse flow around a circular cylinder in the range of Reynolds numbers $740\leq {Re}_{d}\leq 14800$. In research (Prasad and Williamson, 1997) a dependence $f_{SL}/f_{K}=0.0235{Re}_{d}^{0.67}$ was obtained for ${Re}_{d}\leq 10^{5}$. For the studied range of Reynolds numbers ($1600\leq {Re}_{d}\leq 6700$) of the transverse flow around a circular cylinder, the frequency ratio $f_{SL}/f_{V}$ was varied from 3 to 80, which was calculated from the dependences of the studies (Prasad and Williamson, 1997; Rajagopalan and Antonia, 2005). In work (Rajagopalan and Antonia, 2005) it is indicated that the dependence $\sqrt{{Re}_{d}}$ is observed for the pairing process of shear layer vortices, which corresponded to the frequency $f_{SL}/2$, for Reynolds numbers ${Re}_{d}>3\cdot 10^{5}$. When a square cylinder was streamlined, the frequency ratio $f_{SL}/f_{V}$ was changed from 20 to 30 in accordance with the results of (Franceschini et al., 2022).

The results of our studies (Figure 11) showed that formation frequencies of the wake vortices, which corresponded to the Strouhal numbers, are varied from 0.15 to 0.41. The Strouhal numbers, which corresponded to the formation frequencies of high-frequency vortices in the shear layer, varied from 4 to 6. Consequently, the ratio of the frequencies of the shear layer to the frequencies of the wake vortices corresponded to the values $f_{SL}/f_{V}$ =(10–40), which is consistent with the results of the above numerical and experimental studies of the transverse flow around the cylinder. Naturally, the mutual influence of a group of cylinders on the vortex flow leads to changes in the formation frequencies of horseshoe vortices, shear layer vortices, and wake vortices. Such changes are due to the formation of liquid deceleration and acceleration zones inside and about the three-row grillage, the appearance of jet flows, upstream and downstream flows near the streamlined surfaces.

The mutual influence of the vortex and jet flows, the nonlinear nature of their interaction, the amplitude and frequency modulation of the oscillating process lead to the appearance of subharmonics and higher-order harmonics of the fundamental frequencies of the vortex flow in the spectral dependences in Figure 11. The results of the study showed that the frequency of oscillations of the cores of horseshoe vortices is lower than that of the periphery of these vortices. This is due to the fact that small-scale vortices are formed at the periphery of a large-scale vortex, which introduce high-frequency fluctuations into the velocity field and change the frequency content of the spectral dependences. In front of the second row of cylinders, the oscillation frequency of the core and periphery of the large-scale horseshoe vortex decreases as these cylinders are in the wake of the first row of cylinders (see Figures 11a and 11b). The deceleration of the flow behind the cylinders of the first group and the appearance of a return flow in their aft part reduces the intensity and frequency of rotation of the horseshoe vortices that are formed in front of the second group of cylinders.

Similar range of energy-intensive oscillation frequencies of horseshoe-shaped vortex structures observed in numerical and experimental studies. In the work (Kirkil et al., 2006), tonal peaks were observed in the spectra of velocity and pressure fluctuations at frequencies St =(0.1–0.18), which corresponded to bimodal oscillations of the vortex flow in the junction region of the cylinder and plate. In addition, another range of energy-intensive frequencies St =(0.3–0.6) was discovered, which were due to the features of the oscillations of primary and secondary vortices, which formed the horseshoe vortex structure. The results of numerical modeling in the research (Kirkil and Constantinescu, 2015) established the range of energy-intensive frequencies 0.07≤St≤0.65 in the spectrum of velocity fluctuations. The formation of secondary vortices of the horseshoe vortex structure in the separation region of the boundary layer in front of the cylinder was observed with the frequency St =(0.29–0.8). In the experimental work (Dargahi, 1989), discrete components of the spectral power densities of velocity fluctuations were observed in the frequency range 0.058≤St≤1.15, which were also due to bimodal oscillations of the vortex flow and the interaction of vortices in the horseshoe vortex structure in the vicinity of the cylinder. In the work (Wei et al., 2001), increased levels of velocity fluctuations were observed in the frequency range St =(0.15–0.95) in the course of experimental studies of the junction flow with the square cylinder.

The formation and evolution of the vortex and jet flow inside and around a three-row cylindrical grillage has a non-linear and non-stationary character. The spectral analysis of such processes must be carried out both in frequency and in time, taking into account the phase changes of the random process. The use of the short-term Fourier transform makes it possible to obtain the time-frequency characteristics of such processes (Ahmadi et al., 2021; Lantini et al., 2021; Meignen et al., 2021; Smith et al., 2021; Li et al., 2022). The idea of this transformation is simple, namely, the time interval of the existence of a non-stationary process is divided into a number of intervals (time windows). In the process of a short-term Fourier transform, a non-stationary signal is considered as a superposition of short-term stationary signals, and the length of the window determines the time and frequency resolution of the spectrogram or correlogram. Since each time window covers a small area in time, the accuracy of the description of local changes in a random signal increases. The short-time Fourier transform technique was used in our studies and the 20% time window overlap method was applied.

**Figure 12:**
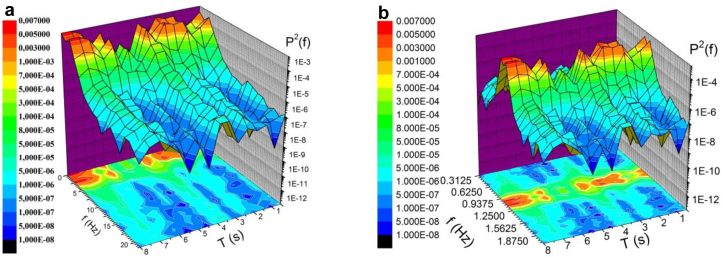
Spectrograms of velocity fluctuations in front of the first central cylinder (a) and in front of the second lateral cylinder (b).

Changes in the spectral power densities of the velocity fluctuations inside the horseshoe vortex structures as a function of time are shown in Figure 12. Figure 12a presents a spectrogram in front of the first central cylinder, and Figure 12b shows a spectrogram in front of the second lateral cylinder. The results were measured at the periphery of the corresponding horseshoe vortex structures for the Reynolds number ${Re}_{d}$ = 2700. It was found that the process of generation and evolution of the horseshoe vortex structures in time had an oscillatory character.

**Figure 13:**
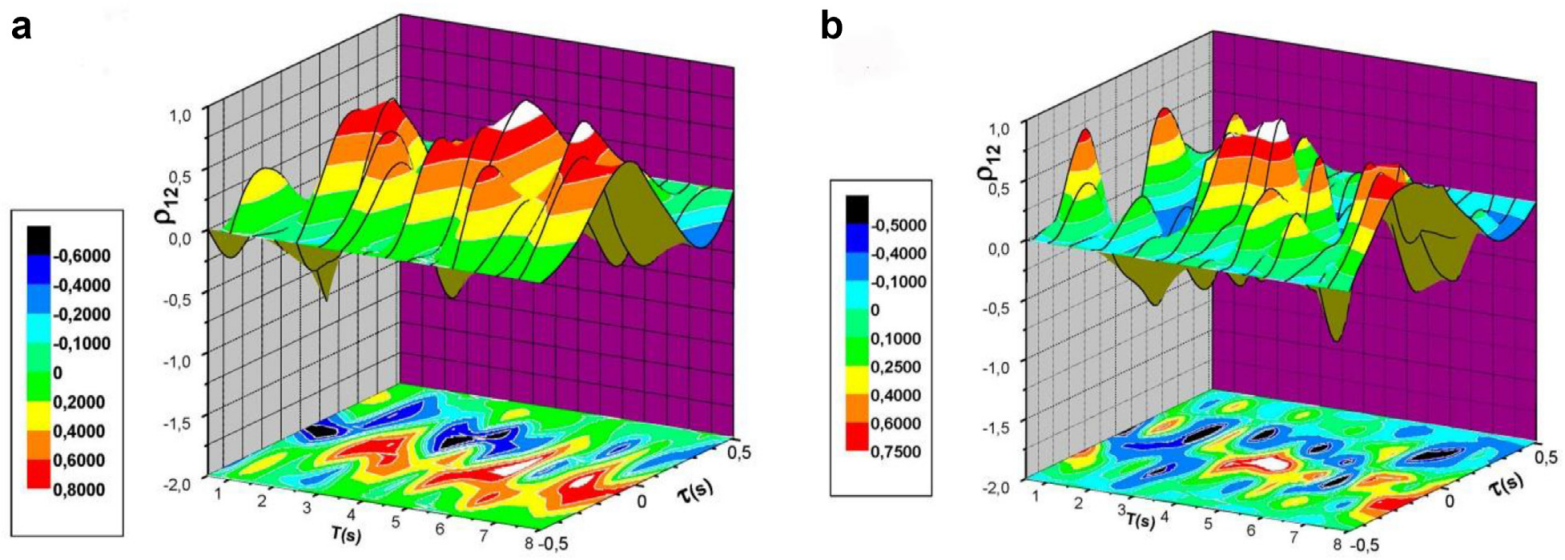
Correlograms of velocity fluctuations in front of the first central cylinder (a) and in front of the second lateral cylinder (b).

The presence of an oscillatory process in the mechanism of generation of the horseshoe vortices was also confirmed in the correlograms shown in Figure 13. Correlograms are cross-correlation functions between two signals of the velocity sensors that were placed at a fixed distance from each other. Figure 13a shows the correlogram of velocity fluctuations at the periphery of the horseshoe vortex structure in front of the first central cylinder of the three-row group, and Figure 13b shows the correlogram of the velocity fluctuations at the periphery of the horseshoe vortex that was formed in front of the second lateral cylinder. It should be noted that the cross-correlation or linear relationship between the velocity fluctuations recorded by the two sensors is quite high. During the study, the maximum values of the cross-correlation were alternated, which corresponded to the periodicity of the process of generation of the horseshoe vortices. However, before the second lateral cylinder, the periodicity is not very pronounced. Here, the randomness of the velocity fluctuation field due to the interaction of the vortex and jet flows became more important.

## 4 Conclusions

Experimental studies were carried out in laboratory conditions in a hydrodynamic channel, where the three-row group of cylinders was installed on the hydraulically smooth rigid surface. The three-row cylindrical group (31 piles with a diameter of 0.027 m) is a model of a bridge support, which was streamlined at a velocity of 0.06 m/s to 0.5 m/s (Reynolds number Re_d_=(1600–6700) and Froude number Fr=(0.04–0.18)). The results of experimental studies showed that the flow around the group of cylinders had a complex unsteady nature, which is due to the interaction of vortex and jet flows with the three-row cylindrical group, which was located on the rigid flat surface.

It was found that horseshoe vortex structures were formed in front of each cylinder, and the system of large-scale horseshoe vortices was generated in front of the entire group of cylinders. Jet flows were appeared between the cylinders of the three-row design, which had the highest intensity in the front and rear groups of the cylinders. The results of flow visualization showed the features of the generation of vortex and jet flows, their evolution and interaction between themselves and the streamlined surface.

Measurements of the velocity field inside and in the vicinity of the front group of three-row cylinders made it possible to determine the integral (in the common frequency band and wavenumbers), spectral and correlation characteristics of the velocity field. Mean, root-mean-square values of velocity and probability density functions of velocity fluctuations integrally displayed the changes in the velocity field in the spatial and temporal domain in the junction area of grillage and plate. The power spectral densities of velocity fluctuations and mutual correlation functions made it possible to study the features of the generation of the velocity fluctuation field in the frequency domain and its interrelationships in space. It has been discovered that the averaged velocity field in the junction flow was multimodal, which was caused by oscillations of horseshoe vortex structures in space and time and their interaction with the unsteady jet flow between the cylinders.

It was revealed that the power spectral densities of the velocity fluctuations were larger at the periphery of quasistable horseshoe vortices than in their cores. The highest levels of the velocity fluctuation spectra were observed in front of the second lateral cylinder of the three-row design, where the interaction of the vortex and jet flows took place. The frequency of oscillations of the cores of horseshoe vortices is lower than that of the periphery of these vortices. Discrete peaks in the spectral levels of velocity fluctuations are found at the frequencies of formation of large-scale wake vortices and the frequencies of formation of small-scale vortex structures of the shear layer, which are due to the Kelvin-Helmholtz instability. It has been established that the frequency of formation of shear layer vortices is (10–40) times higher than the frequency of formation of wake vortices. Spectrograms and correlograms confirmed the periodicity of the formation of horseshoe vortex structures in front of the cylinders of the three-row group structure.

## Declarations

### Author contribution statement

Volodymyr Voskoboinick, Oleksandr Voskoboinyk: Conceived and designed the experiments; Performed the experiments; Analyzed and interpreted the data; Wrote the paper.

Arthur Onyshchenko: Conceived and designed the experiments; Analyzed and interpreted the data; Wrote the paper.

Anastasiia Makarenkova: Performed the experiments; Analyzed and interpreted the data; Contributed reagents, materials, analysis tools or data.

Andrij Voskobiinyk: Performed the experiments; Contributed reagents, materials, analysis tools or data; Wrote the paper.

### Funding statement

This research did not receive any specific grant from funding agencies in the public, commercial, or not-for-profit sectors.

### Data availability statement

Data will be made available on request.

### Declaration of interest's statement

The authors declare no conflict of interest.

### Additional information

No additional information is available for this paper.
